# Intra-Cluster Percolation of Calcium Signals

**DOI:** 10.1371/journal.pone.0008997

**Published:** 2010-02-18

**Authors:** Guillermo Solovey, Silvina Ponce Dawson

**Affiliations:** Departamento de Física, Facultad de Ciencias Exactas y Naturales - Universidad de Buenos Aires, Buenos Aires, Argentina; University of Milano-Bicocca, Italy

## Abstract

Calcium signals are involved in a large variety of physiological processes. Their versatility relies on the diversity of spatio-temporal behaviors that the calcium concentration can display. Calcium entry through inositol 1,4,5-trisphosphate (IP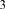) receptors (IP
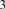
R's) is a key component that participates in both local signals such as “puffs” and in global waves. IP
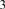
R's are usually organized in clusters on the membrane of the endoplasmic reticulum and their spatial distribution has important effects on the resulting signal. Recent high resolution observations [1] of Ca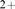 puffs offer a window to study intra-cluster organization. The experiments give the distribution of the number of IP
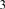
R's that open during each puff without much processing. Here we present a simple model with which we interpret the experimental distribution in terms of two stochastic processes: IP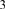 binding and unbinding and Ca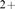-mediated inter-channel coupling. Depending on the parameters of the system, the distribution may be dominated by one or the other process. The transition between both extreme cases is similar to a percolation process. We show how, from an analysis of the experimental distribution, information can be obtained on the relative weight of the two processes. The largest distance over which Ca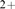-mediated coupling acts and the density of IP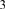-bound IP
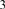
R's of the cluster can also be estimated. The approach allows us to infer properties of the interactions among the channels of the cluster from statistical information on their emergent collective behavior.

## Introduction

The calcium (Ca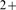) ion is a universal second messenger that is involved in a large number of physiological processes [2]. To this end, cells regulate cytosolic Ca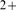 concentration ([Ca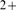]) very precisely. At basal conditions free cytosolic [Ca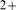] is very low (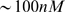). [Ca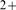] is several orders of magnitude higher in the extracellular medium and in internal reservoirs, such as the endoplasmic reticulum. Different signals can induce the opening of specific Ca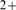 channels located on the plasma membrane or on the membrane of the internal reservoirs leading to local increments of the cytosolic [Ca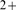] of various durations. This [Ca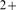] change evokes different end responses depending upon the spatio-temporal distribution of [Ca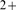]. Thus, it is of interest to measure the latter and how different factors shape it.

One of the Ca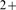 channels involved in intracellular Ca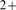 signals is the inositol 1,4,5-trisphosphate (IP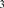) receptor (IP
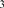
R) which is expressed in many cell types and is located at the surface of intracellular membranes such as the endoplasmic reticulum (ER), the sarcoplasmic reticulum (SR) and the nucleus. The IP
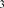
R is biphasically regulated by Ca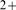, with a bell-shaped open probability as a function of [Ca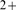]. Kinetic models of the IP
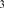
R take this dual effect into account by assuming that the receptor has at least one activating and one inhibitory site such that Ca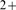 binding to the first one induces channel opening (provided that IP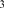 is also bound to the receptor) and Ca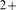 binding to the second one induces channel closing [3]–[5]. Given that the affinity for Ca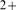 of the activating site is larger than that of the inhibitory site, a local increase of cytosolic Ca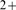 in the vicinity of an IP
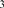
R with IP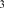 bound induces channel opening first. This leads to a phenomenon called Ca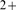-induced Ca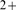-release (CICR) because the Ca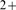 ions released by one channel can in turn trigger the opening of other nearby channels with IP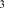 bound. Ca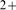 channels are not uniformly distributed in the cell. IP
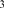
R's, in particular, are usually organized in clusters on the membrane of the ER that are separated by a few microns [6]. These clusters have been estimated to be 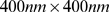 in size in oocytes [7], [8]. The simulations of [7] showed that previous observations could be reproduced assuming that between 25 and 35 IP
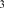
R's opened simultaneously during puffs. A similar estimate was obtained in [8] using a mean-field model that assumed that all channels opened and closed simultaneously. Simulations that include a stochastic description of the individual channel openings and closings, however, show that at most half of the channels with IP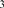 bound are simultaneously open during a puff [8]. This implies that even in clusters with 50 IP
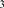
R's with IP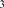 bound, the maximum number of simultaneously open channels is around 20. These results are consistent with observations of Ca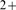 signals in the human neuroblastoma SY5Y cell line in which puffs of up to 20 simultaneously open channels were observed [1]. Measurements performed using patches of the outer nuclear envelope of the DT40 cell line give smaller numbers of IP
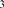
R's in each patch [9]. The non-uniform spatial organization of the IP
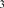
R's together with the channel coupling induced by CICR gives rise to a large variety of intracellular Ca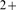 signals that go from very localized ones to waves that propagate throughout the cell [10].

The hierarchy of intracellular Ca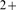 signals that includes Ca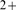 “blips” (Ca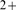 release through a single IP
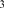
R), “puffs” (Ca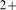 release through several IP
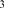
R's in a cluster) and waves that propagate globally across cells by successive cycles of CICR has been observed using fluorescence microscopy and Ca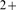 sensitive dyes [10]–[13]. The *Xenopus laevis* oocyte has been frequently used for this purpose because of its relatively large size and because the only Ca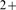 channels that are present on the surface of the ER are IP
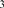
R's. Fluorescent images of these signals obtained with confocal microscopy do not resolve the inner-cluster structure. Therefore, different modeling strategies have been presented in order to determine the properties of the dynamics and spatial organization of IP
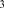
R's within clusters that are compatible with these experimental observations [7], [8], [14], [15]. In particular, in [8], [16] we made the very simple assumption that the number of IP
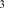
R's that open during the first puff that occurs at a site is given by the number of IP
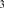
R's with IP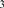 bound. The underlying assumption was that the Ca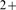 released by the first open channel would induce the opening of all the other IP
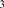
R's of the cluster with IP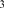 bound. Therefore, if all the clusters had approximately the same number of IP
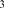
R's and all IP
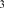
R's were equally sensitive to IP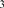, the distribution of the number of channels that opened during a puff could be approximated by a binomial or Poisson distribution [8], provided that the probability that the channels become open were the same immediately before the occurrence of each puff. This last condition would not be satisfied in a non-stationary situation, *e.g.* if the concentrations of the agonists right before the release event differed significantly from puff to puff. It would not hold, in particular, for data containing sequences of puffs that are coupled through CICR or to puffs in which the inhibitory effect of the Ca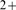 released in a previous event was noticeable, as described in [16]. In oocytes, the latter is only relevant for very long records containing many puffs at a site, which is usually not the case in most experiments. Calcium induced calcium release is also affected by buffers that can trap Ca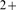 ions as they diffuse. This not only reduces the [Ca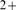] but also alters the rate of Ca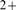 transport [17]. The distances that separate IP
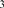
R's within a cluster are very small (10–20nm) [9]. Thus, only large concentrations of very fast buffers could affect Ca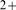-mediated inter-channel coupling in cases with many active channels [18], [19]. The assumption that all the channels with IP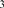 bound participate of the first puff of their site is the simplest way of approaching the complex problem of Ca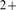-mediated inter-channel communication. Yet, it is applicable as long as the distance between IP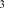-bound channels is not too large. In the present paper we drop this assumption and analyze how Ca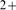-mediated inter-channel coupling affects the distribution of puff sizes. Our approach provides a simple tool to study some of the effects of buffers on the intra-cluster dynamics.

The quantal properties of Ca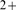 release during puffs have recently been revealed in [1] using total internal reflection fluorescence (TIRF) microscopy in intact mammalian cells of the human neuroblastoma SY5Y cell line. The proximity of IP
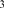
R's to the plasma membrane in this cell type allowed the use of TIRF microscopy in which fluorescence can be elicited in a very small (attoliter) volume. This, together with the use of a fast CCD camera, permitted a much better temporal resolution than the one achieved with confocal microscopy. In this way, abrupt step-wise transitions between fluorescence levels were observed during the falling phase of puffs. Furthermore, many puffs could be elicited at each release site due to the use of a membrane-permeable form of IP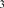 [20]. The authors then inferred that the step-wise transitions between fluorescence levels occurred in multiples of a basic unit that they identified with the amplitude contribution of each channel at the site [1]. Using this relationship they could readily obtain the distribution of the number of channels that open during a puff. Given that there is a large variability among cluster sites, they analyzed the subset of events that occur in clusters with a similar number of IP
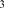
R's. The authors did not find any sign of an inhibiting effect of the Ca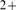 released in their records. In spite of that and even constraining the data set as mentioned before, they found that a Poisson distribution failed to reproduce the observed histogram of event sizes particularly in the region of small events (*i.e.*, puffs with very few open channels). They could approximately describe the distribution with a model that assumes a weak cooperativity among channels. Inter-channel cooperativity is mediated by the Ca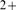 released through an open IP
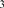
R that subsequently diffuses to a neighboring channel. Thus, the distance between channels is a key factor that regulates the cooperativity strength [21]. The approach of [1], however, does not take space into account.

In the present paper we introduce a simple model that takes into account both the stochasticity due to IP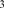 binding and the distance-dependent Ca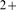-mediated cooperativity. It can reproduce the event size distribution reported in [1] for events involving any number of open channels. The distribution obtained with our model approaches a binomial or Poisson distribution as the cooperativity strength increases so that the opening of one IP
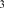
R induces the opening of all other IP
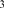
R's with IP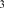 bound. This transition from Ca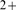-dominated to IP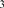-binding dominated stochasticity is similar to a percolation transition. It also occurs if the number of IP
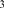
R's with IP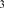 bound increases. Therefore, the transition can be reflected on the distribution of the number of IP
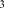
R's that open at a given release site.

Percolation in connection with Ca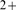 signals has been invoked to explain the transition from abortive to propagating waves in cells [22]–[24]. Our paper is the first to identify two limiting regimes of the intra-cluster dynamics that underlies puffs and to characterize the change between them as a percolation transition. Furthermore, we show how information on the transition between both regimes (the IP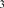-binding and the Ca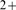 dominated behaviors) can be extracted from the distribution of the number of IP
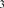
R's that open during a puff. Knowledge on this transition can, in turn, yield information on the largest distance over which Ca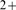-mediated cooperativity acts and on the mean density of IP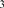-bound IP
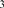
R's of the clusters. In this way, we can estimate biophysical parameters that affect the intra-cluster dynamics from statistical information on the emergent collective behavior of the channels of the cluster.

The aim of the simple model that we introduce in this paper is to characterize the basic mechanisms that shape the distribution of the number of channels that open during puffs. In particular, we identify the competition between two stochastic processes as the main determinant of the form of the distribution. Therefore, an analysis of this form may give information on the relative weight of the two competing processes. The model does not include a detailed description of the dynamics that takes place during or between events. For some time most models of intracellular Ca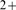 dynamics were deterministic (see *e.g.* [25]). The observation of local signals such as puffs led to the development of several models that included a stochastic description of Ca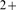 release [14], [15], [26], [27] or of the spatial location of the IP
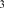
R's [28]. It is currently clear that stochastic effects are not only relevant for local release events but are a fundamental aspect of the Ca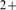 dynamics for the full range of observed signals, including waves [29]–[32]. More information on stochastic models of Ca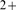 signals can be found in a recent focus issue on the subject [33]. Simulations of these stochastic dynamic models could be used to probe the main findings of the present paper.

## Results

### The Model

We introduce here a simple model to describe the distribution of puff sizes that occur at sites with similar numbers of IP
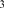
R's. The model is simple in the sense that it does not include a detailed description of the dynamics of the individual channel openings and closings or of IP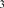 or Ca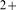 binding and unbinding. However, it does include the stochasticity associated to IP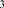 binding and channel coupling via CICR. Given the estimates of [8], the model assumes that clusters occupy a fixed size region (more specifically, a circle of radius, 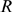) and that 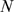 IP
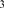
R's are randomly distributed over the cluster region with uniform probability. Each IP
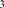
R of the cluster has a probability 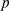 of having IP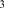 bound. 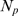 is the random variable that represents the number of available IP
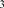
R's (*i.e.*, of IP
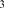
R's with IP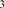 bound) of the cluster before a puff starts. The distribution of this variable is a binomial of parameters 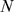 and 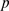, that can be approximated by a Poisson distribution of parameter 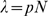 for 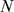 large and 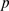 small enough (for example, with 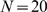 and 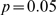 the absolute difference between both cumulative distributions is lower than 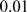 for each 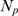). The model considers that if an IP
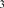
R with IP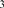 bound becomes open and Ca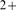 starts to flow through its pore all other IP
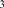
R's with IP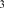 bound that are within a distance, 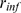, of the open IP
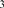
R will also become open. These newly opened IP
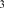
R's in turn trigger the opening of new IP
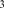
R's with IP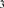 bound that are within the distance, 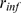, from an open one. This scheme triggers a cascade of openings that stops when there are no more available IP
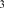
R's within the radius of influence (*i.e.*, the distance 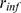) of any open IP
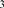
R. This cascade determines the number, 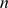, of channels that open during a puff. We call 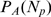 the probability that there are 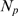 available IP
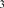
R's (with IP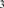 bound) in a cluster and 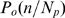 the conditional probability that 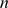 channels open during an event given that there are 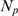 with IP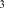 bound in the cluster. Given that we are interested in the distribution of event sizes, we only consider the situations for which 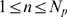. Therefore, we renormalize the probabilities so that 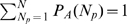 and 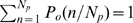. In this way, 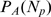 is a binomial or a Poisson distribution divided by one minus the probability that there are not IP
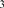
R's with IP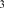 bound in the cluster. Using these renormalized versions of 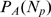 and 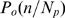, the probability, 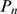, of having a puff with 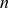 open channels is given by:
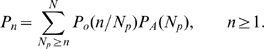
(1)

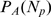
 is approximated by a Poisson distribution of parameter 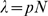 when 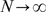. 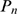 can be readily compared with distributions obtained from experimental observations as the one displayed in Fig. 4D of [1].

### Factors That Shape the Distribution of Event Sizes

The two stochastic components of the model are evident in the expression of 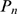. 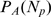 reflects the stochasticity of IP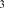 binding and 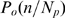 the one due to inter-channel coupling via CICR. The relative weight of both factors on the resulting 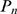 depends on the relationship between two typical lengthscales of the problem: the radius of influence, 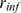 (the maximum distance between channels at which CICR still works) and the mean distance between channels with IP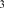 bound, 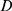, which is a random variable that can be computed in terms of 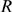 and the number of IP
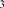
R's with IP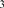 bound, 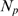, as:
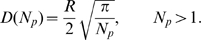
(2)

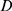
 can take values between 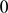 and 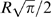. Closely related to 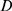 is the density of available IP
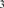
R's which is given by:
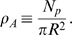
(3)

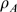
 and 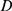 are related by: 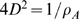.

The relationship between 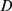 and 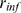 determines the relative weight of both stochastic components on 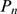. In particular, if 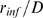 is very large, the opening of any channel of the cluster will eventually lead to the opening of all other available channels. If such a situation holds for most events, then 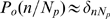 and 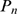 will mainly be determined by the stochastic component due to IP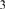 binding, *i.e.*, 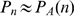. If, for most events, 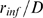 is very small, then most of 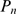 will be concentrated near 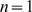, regardless of how many available IP
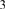
R's there are in each realization. We will refer to both extreme behaviors as IP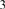 or Ca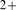 limited. Depending on the parameters of the model (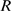, 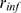, 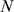, and 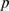), one or the other situation is favored. However, in many situations one or the other behavior is favored depending on the value of 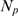, *i.e.*, on the realization. In those cases, the dominant stochastic component of 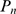 depends on the value of 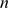.

**Figure 1:**
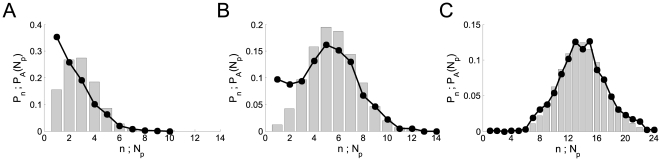
Distribution of puff sizes: transition between Ca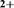-dominated to IP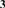-binding dominated stochasticity. Solid circles: distribution of puff sizes, 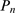, obtained with our model for 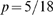, 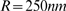, 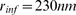 and three values of 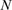: 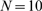 (A), 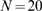 (B) and 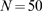 (C). Histograms (in grey): corresponding distributions of available channels, 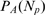 for the same parameter values. All distributions were computed from 1000 realizations for each set of parameters.

We first illustrate how the distribution, 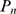, varies with the number of IP
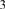
R's of the cluster, 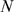, while all other parameters are fixed. As 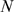 increases, the most likely values that 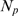 can take on also increase. This means that it is more probable to have more available IP
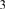
R's at any given instance. On the other hand, since the spatial dimensions of the cluster are unchanged (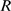 is fixed) the mean distance between available IP
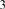
R's, 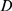, is more likely to be smaller (see Eq. 2). Given that the typical distance for CICR to occur, 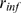, is also fixed, it is more probable that 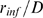 be larger. Therefore, 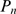 approaches 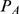 as 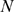 is increased. This is illustrated in Fig. 1 where we have plotted the distributions 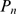 (solid circles) and 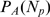 (bars) obtained with 1000 realizations of our model using 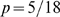, 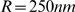, 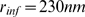 and three values of 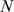. In A, 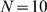, the number of available channels is small for most realizations (its mean value is 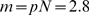) so that 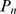 is dominated by inter-channel Ca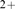-coupling and concentrated around small values of 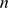. In C, 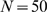, the number of available channels is large for most realizations (its mean value is 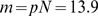) so that 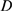 is typically smaller than 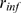 (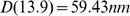). In this case, 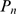 is dominated by the IP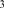-binding stochasticity and almost indistinguishable from the distribution of available channels, 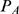. The example of Fig. 1 B corresponds to a situation in between these two extreme cases with 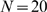. We can observe how, as the number of available channels is more likely to be larger, 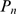 approaches 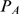. We also observe that for 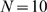 and 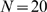, 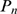 and 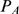 differ mainly in the region of small values of 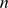. This occurs because it is difficult for one open channel to induce the opening of another one if the mean inter-channel distance is large. Thus, if 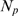 is small it is very rare that all available channels become open. In this way, the relative frequency of small events becomes larger than the fraction of instances with a small number of available channels.

**Figure 2:**
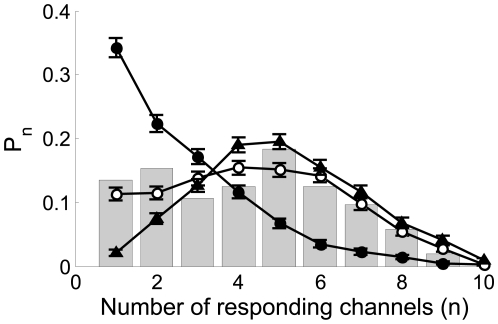
Distribution of puff sizes: change of behavior with the radius of influence and comparison with observations. We show the probabiliy, 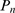, of having a puff with 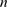 open channels obtained with our model for 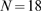, 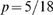, 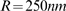 and 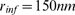 (solid circles), 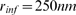 (open circles) and 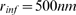 (triangles). Each curve corresponds to 500 realizations of the model. We observe a transition from a Ca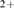-dominated to a IP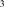-binding dominated stochasticity distribution as 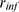 increases. Superimposed with bars: experimental data taken from Fig. 4D of [1].

A transition from Ca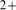-dominated to IP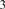-binding dominated stochasticity also occurs as 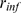 is increased, while all other parameters are fixed. In this case, 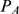 remains unchanged and so does the mean distance between available IP
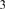
R's, 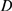. By changing 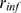 it is possible to go from a situation in which 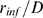 is small for most events and 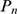 is Ca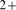-limited to a situation in which 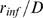 is large and 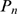 is IP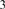-binding limited. This is illustrated in Fig. 2 where we have plotted the distribution of event sizes that we obtain with our model for three different values of 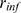. For 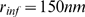, the distribution is Ca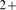-coupling limited and is concentrated around 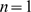. As 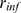 is increased, the relative frequency of events with small 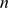 decreases. For 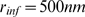, the distribution is IP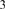-binding limited. In this example, 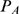 is well approximated by a Poisson distribution of parameter 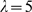 (data not shown). The situation in between these extreme cases corresponds to 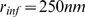 and is able to reproduce reasonably well the experimental distribution of Fig. 4D of [1] (superimposed with bars in Fig. 2).

**Figure 3:**
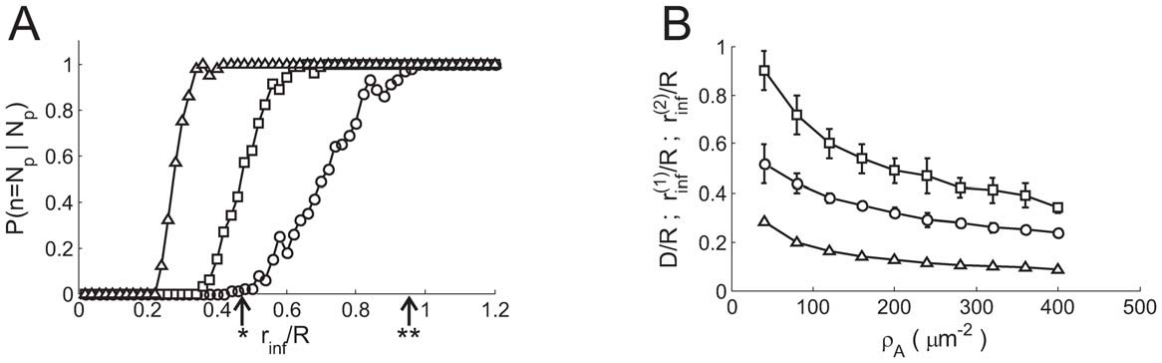
Percolation transition: when all available channels open during a puff. A: Probability that all available IP
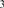
R's become open, 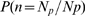, as a function of the dimensionless radius of influence, 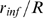, for 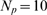 (circles), 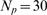 (squares) and 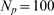 (triangles). B: 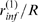 (circles), 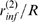 (squares) and 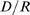 (triangles) as functions of 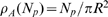. The values of 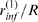 and 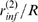 for the case with 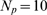 are indicated in A with one and two asterisks, respectively.

In the Ca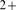 limited behavior the number of open channels, 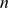, is small for most events, regardless of the value of 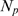. This implies 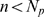 for almost all events. In the IP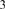-binding limited behavior all available IP
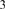
R's become open (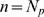 in most cases). Therefore, in order to analyze the transition between the Ca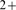-dominated to IP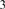-binding dominated stochasticity, we study how often events occur for which all available IP
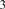
R's become open. This happens trivially for events with 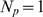. Here we are interested in situations with 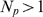. To this end, we compute numerically the probability that all available IP
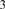
R's, 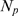, become open, 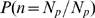, which is a function of 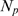 and of only one independent parameter, the dimensionless radius of influence, 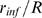, (see Methods). We plot in Fig. 3 A 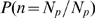 as a function of 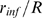, for 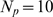 (circles), 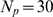 (squares) and 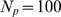 (triangles). As expected, 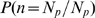 is an increasing function of 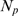 for each value of 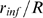. We also observe that 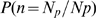 is an increasing (sigmoidal-like) function of 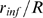 that goes from 0 (*i.e.* 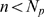 in almost all cases, which corresponds to Ca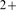-dominated stochasticity) to 1 (*i.e.* 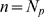 in almost all cases, which corresponds to IP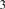-binding dominated stochasticity) and that such transition occurs over a smaller interval of 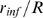 values the larger 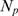 is.

We can think of the Ca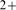-limited and the IP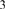-binding limited situations as two phases and the transition between them as a phase transition in the limit of very large 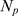. This percolation-like transition occurs at a well defined value of 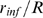 in this limit. For finite values of 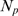 we introduce two quantities, 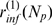 and 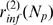, that determine the type of regime that we can expect (Ca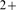-limited if 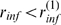 or IP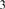-binding limited if 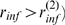) for each value of 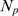 (see Methods). The arrows in Fig. 3 A indicate the values of 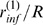 (*) and 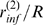 (**) for the 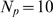 case. We show in Fig. 3 B plots of 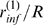, 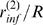 and 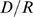 as functions of 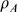 (Eq. (3)). It is important to note that these curves are the same, regardless of the specific parameter values of the model. We observe that all of them are decreasing functions of 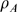 or, equivalently, of 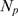. 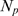 is a stochastic variable that changes from realization to realization. Therefore, even for a given cluster (characterized by fixed values of 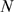, 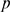 and 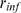) 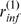 and 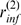 may take on different values depending on the realization. In this way, depending on 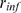 and the values that 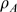 may take on, a subset of the events that occur at a cluster may be IP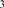-binding limited (those for which 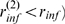) while others are not. An analogous situation may hold regarding the Ca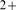-limited behavior. Furthermore, for some clusters, the Ca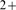-limited condition may hold for some events and the IP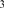-binding limited for others. If the parameters 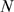, 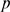, 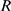 and 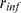 are such that most realizations satisfy 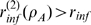, then most events will be IP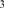-binding limited. This happens if 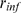 or 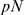 are large enough, in which case the distribution of event sizes, 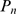, approaches the distribution of available channels, 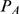.

### Observing Percolation as a Function of Event Size

The results of Fig. 3 B imply that there are clusters that can display different types of behaviors depending on the event. For these clusters, we expect to find, in their distribution, 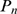, a trace of the transition to the limiting behavior that they can display. Here we are interested in the percolation transition, *i.e.*, the transition to the IP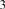-binding dominated stochasticity. As already discussed, the larger 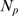 the more likely it is that all IP
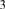
R's become open during the puff (see Fig. 3 A). Thus, the transition to the IP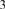-binding dominated stochasticity should occur as 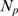 and, consequently, 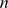 become larger. To study this transition we consider a cluster with fixed parameters 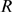, 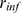, 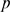 and 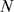 (or 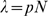 in the Poisson limit) and define 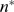 as the minimum value of 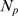 such that 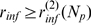. The definition of 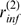 is based on the conditional probability, 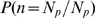, which is independent of 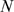. In cases with finite 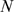, 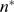 is meaningful provided that it be smaller than 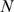. Since 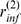 decreases with 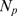 (see Fig. 3 B), taking into account the definitions of 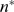 and of 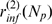 (see Methods) we conclude that 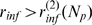 and 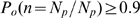 for all 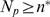. Thus, we can approximate:
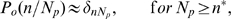
(4)with less than 10% error. Inserting this approximation in Eq. (1) we obtain:
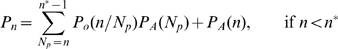
(5)

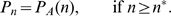
(6)We then conclude that the 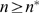 tail of 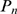 corresponds to IP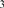-binding dominated events. Therefore, it should be possible to approximate it by a (renormalized) binomial (provided that 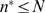) or Poisson distribution in the region of large 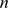. The left border of this IP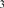 dominated behavior, 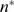, gives information on 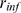, *i.e.* on the maximum distance for which CICR-coupling can work effectively. Therefore, it should be possible to estimate 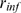 by analyzing 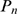, *i.e.*, to infer a biophysical parameter that characterizes the intra-cluster dynamics from statistical information on the emergent collective behavior of the channels of the cluster.

### Determining Intra-Cluster Properties from Observations of the Cluster as a Whole

We now discuss how we can estimate 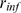 from an experimental distribution of event sizes, 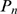. For the sake of simplicity, we assume that 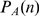 can be approximated by a renormalized Poisson distribution, 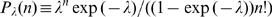, of unknown parameter 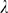. The goal of this section is to provide a way to estimate 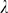 and 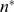, the value of 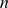 at which 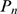 and 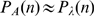 depart from one another (see Eq. (6)). Once 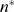 is inferred, we estimate 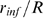 as 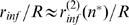 using the function displayed in Fig. 3 B. To this end, we focus on the large 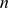 tails of 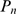 and 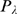 by computing the complementary cumulative distribution functions:
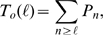
(7)

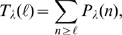
(8)for 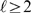. Given that 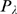 is proportional to a Poisson distribution, there is an analytic expression for 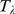. Namely, 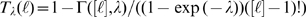, where 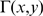 is the incomplete 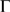 function and 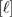 is the integer part of 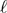. If the cluster is such that 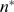 exists so that 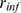 is larger than 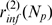 for 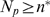 and it is smaller otherwise, then, according to the calculation of the previous section, 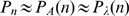 for 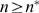. Therefore, the complementary cumulative distribution functions of Eqs. (7)–(8) also satisfy 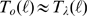 for 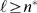.

We now describe how to estimate 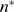 and 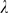. The aim is to obtain a (renormalized) Poisson distribution, 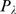 that can approximate 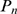 in the large 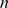 region. If we find it, we assume that it is a good approximation of the distribution of available channels, 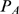. As illustrated in Fig. 1, the mean value, 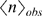 that is obtained using the experimental distribution, 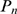, is smaller than the one that would be obtained if 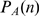 was used instead. On the other hand, if 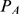 is a good approximation of 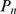 in the large 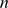 region, then the mean value obtained with 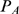 should be smaller than the size of the largest observed event, 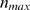. This implies that
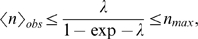
(9)if 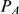 can be approximated by a renormalized Poisson distribution of parameter 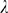. Therefore, we look for the best 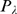 within a finite set of renormalized Poisson distributions of parameters 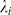 satisfying (9). In order to estimate 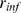 from the observations the relevant quantity that we need to obtain is 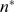, which is an integer. For this purpose, it is possible to use a rather coarse grid of 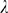 values within the interval defined in (9). In particular, we have mainly used integer values of 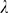 obtaining good results. Once the values 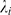 are chosen, we compute the complementary cumulative distribution functions, 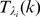 given by (8) for each 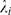 and 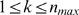. We then calculate the error of approximating 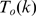 by 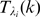 over the interval 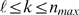 as a function of 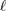:
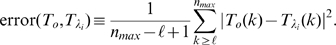
(10)We set a threshold for the error, 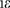, and choose 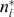 for each 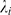 as the smallest value of 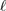 for which 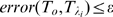. Finally, we choose the best 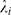 as the one with the smallest 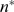.

**Figure 4:**
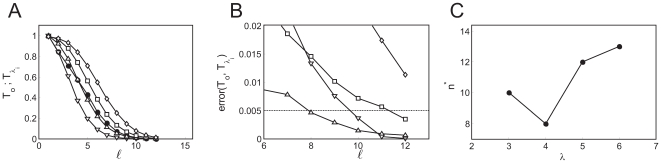
Change of behavior with event size. A: 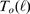 for data obtained with our model using 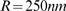, 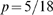, 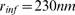 and 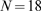 (solid circles). Complementary cumulative Poisson distributions, 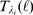, for 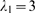 (inverted triangles), 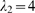 (triangles), 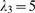 (squares), 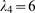 (rhombes). B: Error of approximating 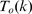 by the various 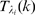 for 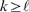 (see text for definition) as a function of 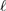. Symbols are the same as in A. From this figure we choose 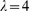 as the one that provides the best fit to the tail of 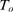. The error in the 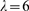 case is larger than 0.02 in most cases and falls outside the region displayed in the figure. C: 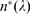 for the four values of 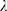 that we tested. We see that 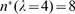.

The procedure is illustrated in Fig. 4 where the “experimental” distribution comes from a simulation of our model with 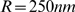, 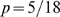, 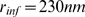 and 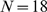. In this case, 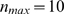. We show in Fig. 4 A the complementary cumulative distribution functions and in Fig. 4 B the errors for the values of 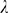 that we have considered: 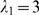 (inverted triangles), 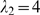 (triangles), 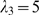 (squares) and 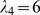 (rhombes). Larger values of 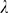 give very bad approximations and are not shown. We show in Fig. 4 C the values, 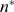, obtained for each 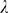 using the threshold, 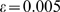 (shown with a horizontal line in Fig. 4 B). In this example, the best value is 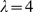 for which 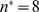. We estimate the density of IP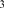-bound IP
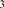
R's at which the departure between the experimental and the Poisson distribution occurs as 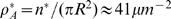, where we have used 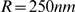. Using the 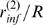 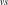 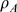 relationship displayed in Fig. 3 B, we estimate 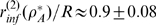 from which we get 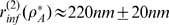. This provides an estimate of the radius of influence which compares very well with the value that was used to generate the data, 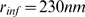. Using the same procedure, we analyzed the data presented in Fig. 4D of [1] and obtained 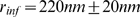 assuming 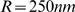.

## Discussion

Intracellular Ca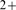 signals are built from localized release events in which Ca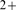 enters the cytosol through one or several channels. Ca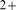 release from the endoplasmic reticulum through IP
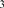
R's is a key component of the Ca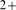 signaling toolkit in many cell types. IP
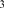
R's are Ca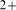 channels that need to bind IP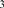 and Ca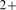 to become open and are usually organized in clusters on the membrane of the endoplasmic reticulum. The intra-cluster organization and the interactions of the channels within it affect the dynamics and extent of the signals. Therefore, their study is a matter of active research.

Recent experiments [1] that use super-resolution optical techniques are providing detailed data on elementary IP
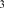
R-mediated Ca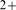 release events in mammalian cells. In the experiments, the number of IP
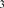
R- Ca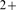-channels that open during each event can be inferred from the observed puff amplitudes without much processing. The observations of [1] showed that the variability among clusters affected the shape of the event size distribution, 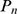. In order to get rid of this variability, the distribution coming from sites with similar properties was computed in [1]. The distribution, 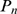, obtained in this way was not Poisson, as we might have expected if the number of channels that opened during each event was proportional to the number of IP
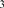
R's with IP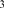 bound in the cluster [8]. The authors of [1] could reproduce 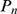 approximately (for events larger than a certain size) assuming a weak cooperativity among channels. Namely, they assumed that the probability that a channel became open scaled as some power of the number of open channels and obtained that the exponent was 1/3 from a fit to the data. The rationale for the cooperativity assumption relied on the fact that the IP
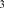
R's of a cluster may be coupled via CICR induced by the Ca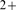 ions that travel from an open IP
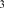
R to a neighboring one. The model of [1], however, did not take space into account and did not provide a mechanistic explanation for the obtained scaling.

In this paper we have presented a simple model that includes a description of the intra-cluster spatial organization with which we can reproduce the observed distribution over all event sizes. In the model the distribution, 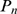, is the result of the competition of two stochastic processes: IP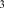 binding and distance-dependent CICR. The model assumes a stationarity condition, namely, that the agonists concentration at the release site is the same immediately before the occurrence of each puff. This condition holds as long as puffs are independent of one another. This is consistent with the observations reported in [1] where cluster coupling was prevented using the slow Ca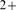 buffer EGTA and where IP
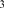
R- Ca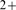-inhibition does not play a significant role. In any case, our model is adequate to describe the distribution of first event sizes that occur at each cluster before Ca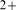 can exert any inhibiting effect.

There are two limiting cases in which one of the two stochastic processes considered in the model is the main determinant of the distribution shape. If the mean distance between IP
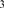
R's with IP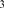 bound in the cluster is much smaller than the typical distance of inter-channel coupling due to CICR for most events, the distribution is IP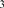-binding limited and it can be approximated by a binomial or Poisson distribution. In the opposite case, CICR dominates and the distribution is peaked around 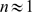. The Ca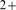-limited and the IP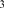-binding limited situations can be thought of as two phases and the transition between them as a percolation-like transition in the limit in which the number of IP
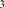
R's with IP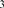 bound, 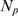, is very large. This interpretation of the factors that shape the observed distribution can be tested with simulations of some of the stochastic models of intracellular Ca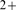 signals reported in the literature (see *e.g.* [33] and references therein). They can also be tested experimentally. One possibility is to change the most likely values of 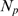 by changing the amount of IP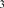 that is photo-released in the cell. An alternative option is to analyze 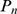 for events coming from clusters that give rise, on average, to larger events. According to the model, the distribution should approach a binomial or Poisson distribution as the mean value of 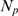 becomes larger while other parameters remain the same. Another way to affect the balance between both stochastic components is to disrupt Ca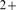-mediated inter-channel coupling by means of a fast buffer such as BAPTA.

Given that 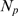 is a stochastic variable that varies from event to event, the transition between the Ca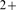-dominated and IP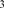-binding dominated stochasticity described by the model may be reflected in the way that 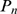 depends on the event size, 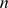. In fact, we have used this property to show how a fingerprint of this transition may be encountered in 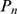 and how information on the inter-channel coupling distance may be extracted from it. This means that a parameter that characterizes the communication between pairs of channels can be estimated from statistical information on the emergent collective behavior of the channels of the cluster. This information could be used to analyze the effect of buffers on the intra-cluster dynamics, a matter that is of active current research [19], [34]. Our model provides a simple tool with which this effect can be analyzed in experiments.

## Methods

Each term of the sum that defines Eq. (1) is the product of two functions. We have an analytic expression for one of them, 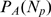, but not for 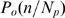. Thus, we compute 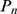 numerically performing realizations of the model with fixed values of 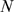, 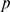, 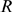 and 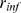. The location of the channels within the cluster and which of them have IP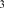 bound vary among realizations and are chosen randomly (see Results). We only keep realizations with 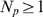. Once we have the spatial distribution of available IP
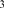
R's, we start each event by picking at random one of the IP
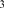
R's with IP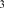 bound and assume it is open. If 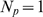, we assume it gives rise to an event with 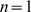. By changing the values of 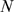, 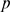, 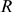 and 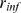 we analyze how 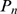 varies with them. In this way we can determine the values of the parameters that best reproduce the experimental observations. 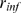 could be measured in units of the cluster spatial extent, 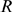, in which case we would get rid of one parameter of the problem, 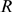. We keep it to make a connection with the experimental data. However, it is important to note that the number of independent parameters of the model is 3, for finite 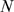 and 2 in the limit in which 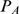 can be approximated by a Poisson distribution.

For each value, 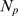, of available IP
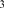
R's, we estimate the fraction of events such that the 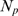 IP
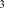
R's become open. This fraction is one for 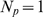. For 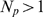, we compute the probability that all available IP
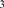
R's become open, 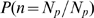, numerically, performing 500 stochastic realizations of our model for each of which we fix the value of 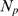 *a priori*. Namely, we fix at the beginning the values of 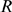, 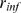 and 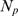 and then pick 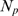 locations at random over the circle where we assume there are available IP
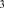
R's. From there on, the model goes on as before, generating the cascade of openings that determines 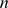. The distribution of events with 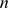 open channels for each value of 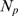 gives 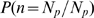. This function of 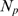 depends on only one independent parameter, 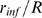. As expected, it is an increasing function of 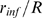 (see Fig. 3 A).

We define two quantities, 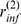 and 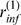, which are values of 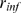 for which 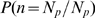 is either close to 1 or to 0, respectively. We compute them as follows. We first calculate a lower bound for 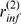 as the minimum value of 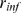 such that, if 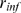 is larger than this lower bound, then 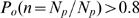. We calculate an upper bound for 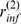 as the minimum value of 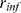 for which 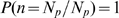. Then, we compute 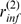 as the mean between these two bounds. We assume that the distance between the bounds is the error with which 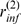 can be determined. We proceed analogously in the case of 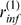, but in this case the lower bound is the largest value of 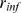 for which 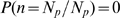 and the upper bound is the maximum value of 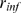 for which 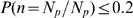. We compute 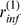 and 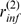 in this way using the numerical estimations of 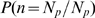 for various values of 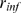.
